# Effect of Low-Temperature Oxygen Plasma Treatment of Titanium Alloy Surface on Tannic Acid Coating Deposition

**DOI:** 10.3390/ma17051065

**Published:** 2024-02-26

**Authors:** Mariusz Winiecki, Magdalena Stepczyńska, Krzysztof Moraczewski, Lukasz Skowronski, Marek Trzcinski, Tomasz Rerek, Rafał Malinowski

**Affiliations:** 1 Department of Constructional Materials and Biomaterials, Faculty of Materials Engineering, Kazimierz Wielki University, Chodkiewicza 30, 85-064 Bydgoszcz, Poland; 2 Department of Polymer Materials Engineering, Faculty of Materials Engineering, Kazimierz Wielki University, Chodkiewicza 30, 85-064 Bydgoszcz, Poland; m.stepczynska@ukw.edu.pl (M.S.); kmm@ukw.edu.pl (K.M.); 3 Division of Surface Science, Faculty of Chemical Technology and Engineering, Bydgoszcz University of Science and Technology, Kaliskiego 7, 85-796 Bydgoszcz, Poland; lukasz.skowronski@pbs.edu.pl (L.S.); marekt@pbs.edu.pl (M.T.); tomasz.rerek@pbs.edu.pl (T.R.); 4 Łukasiewicz Research Network—Institute for Engineering of Polymer Materials and Dyes, Marii Skłodowskiej-Curie 55, 87-100 Torun, Poland

**Keywords:** titanium alloy, surface modification, low-temperature plasma, tannic acid, coating

## Abstract

In this study, the effect of low-temperature oxygen plasma treatment with various powers of a titanium alloy surface on the structural and morphological properties of a substrate and the deposition of a tannic acid coating was investigated. The surface characteristics of the titanium alloy were evaluated by X-ray photoelectron spectroscopy (XPS), scanning electron microscopy (SEM), atomic force microscopy (AFM), and contact angle measurements. Following this, the tannic acid coatings were deposited on the titanium alloy substrates and the structural and morphological properties of the tannic acid coatings deposited were subject to characterization by XPS, SEM, and spectroscopic ellipsometry (SE) measurements. The results show that the low-temperature oxygen plasma treatment of titanium alloys leads to the formation of titanium dioxides that contain –OH groups on the surface being accompanied by a reduction in carbon, which imparts hydrophilicity to the titanium substrate, and the effect increases with the applied plasma power. The performed titanium alloy substrate modification translates into the quality of the deposited tannic acid coating standing out by higher uniformity of the coating, lower number of defects indicating delamination or incomplete bonding of the coating with the substrate, lower number of cracks, thinner cracks, and higher thickness of the tannic acid coatings compared to the non-treated titanium alloy substrate. A similar effect is observed as the applied plasma power increases.

## 1. Introduction

Titanium (Ti) alloys are widely used as endosseous implant material due to their excellent biocompatibility and their ability to allow bone–implant integration [1]. Despite its high biocompatibility, an untreated Ti alloy is not particularly bioactive because of a thin native oxide layer. To increase its bioactivity, it requires surface modification to improve wettability and cell adhesion and proliferation, resulting in the achievement of robust osseointegration between the Ti alloy implant and the surrounding bone [2,3]. A variety of surface modification methods allow functionalization of the surface of endosseous implants, both at the micro and nano scales, allowing for an enhanced cell culture response [4,5,6] and/or showing the potential to inhibit adhesion or kill microbial cells that contact the surface [7,8].

Plasma surface modification is known to be an effective and economical surface treatment technique for many materials. Because it is a highly unusual and reactive chemical environment in which many plasma surface reactions occur, its use can result in changes in a variety of surface characteristics, for example, chemical, tribological, electrical, optical, biological, and mechanical [9]. Its use includes, but is not limited to, surface decontamination (cleaning/disinfection/sterilization) [10,11,12], surface etching [13] and micro-patterning [14,15], thin films, and coating deposition [16]. Plasma treatment allows the enhancement of the performance of biomaterials since their surface properties can be selectively modified to make them medically biocompatible. Plasma surface modification strategies allow one to tailor the surface topography and/or surface chemistry of biomaterials and derive their biocompatibility [17] and antibacterial properties [18,19,20,21] or improve osseointegration [22,23,24].

Low-temperature plasma (LTP), also called cold plasma, which contains high-energy electrons and a large number of excited atoms, molecules, ions, free radicals, and other active substances, can produce different surface treatment effects under different reaction conditions [25]. The possible known changes in material surface properties due to the applied LTP modification are cleaning the organic pollutants on the material surface; thin film deposition on the material surface; etching, i.e., loss of material surface particles; and modification/functionalization, like improving the hydrophilicity of the material surface and the material surface activation, as LTP can promote the production of carbonyl, carboxyl, and oxygen atoms and other groups on the material surface [24,26]. The modification of the surface of titanium by low-temperature plasma can connect chemical functional groups to the surface [27], resulting in improved surface wettability and enhancing its adhesive properties [28]. Physicochemical changes have been observed, including changes in the content of hydrocarbon and functional hydroxyl groups, as well as surface free energy levels [29,30]. Cold plasma activates the titanium surface, not only affecting its specific biological performance directly but also facilitating coating deposition, which can be applied simultaneously (plasma-assisted coating deposition) and sequentially (plasma activation followed by coating deposition) [31].

Current research and development trends in the field of implant surface engineering aim to improve functional properties by producing coatings that trigger the desired biological responses and result in rapid and permanent implant fixations within the native bone. Tannic acid (TA), a natural polyphenol extracted from plants with a strong affinity for various surfaces, possesses superior properties to serve as a functional coating [32]. Bioinspired TA coatings exhibit antioxidant, antibacterial, antimutagenic, and antigenic activities [33,34], as well as having a high potential to be harnessed for local delivery of active molecules in the peri-implant environment to induce anti-inflammatory effects on various cell types in vitro or modulate osteoblasts and osteoclasts in vitro [35,36,37,38,39].

In this study, the effect of low-temperature oxygen plasma treatment with various powers of a titanium alloy surface on the structural and morphological properties of a substrate and the deposition of a tannic acid coating was investigated.

## 2. Materials and Methods

### 2.1. Sample Modification

Samples in the shape of discs 8 mm in diameter and 1 mm in thickness were machined from a Ti-6Al-4V (Grade 5) rod (Bibus Metals, Dąbrowa, Poland). The discs were polished with abrasive SiC paper (600, 1200, and 2000 grids) and divided into three groups. Two groups of discs were subjected to low-temperature plasma (LTP) treatment and the reference group remained untreated. The plasma modification was performed using the Femto Plasma Generator (Diener electronic GmbH & Co. KG, Ebhausen, Germany) with a nominal power of 100 W. The discs were placed in a plasma generator chamber on a metal slab and exposed to plasma discharge. The samples were treated for 10 min at room temperature with an O_2_ plasma for 10 min with a gas flow rate of 15 mL/min and a chamber pressure of 0.1–1 mbar. The plasma powers of 40 W and 80 W were employed, and depending on the plasma power, samples are referred to as Ti40 and Ti80, and the reference sample is denoted as Ti0.

Subsequently, surface modification of the samples specified above with tannic acid coatings was performed. In the modification process, tannic acid C_76_H_52_O_46_ with a molecular weight of 1701.20 g/mol (Merck KGaA, Darmstadt, Germany) was used. Tannic acid aqueous solutions were prepared in two different concentrations (2% and 5%). The Ti discs were placed in the prepared solutions and left for a 24 h time period at room temperature to deposit the tannic acid coating on the surface of the titanium discs. After 24 h, the discs were removed from the acid solutions and dried for 24 h at 40 °C. Depending on the concentration of the tannic acid solutions used, the samples are referred as Ti0-Ta2, Ti40-Ta2, Ti80-Ta2, Ti0-Ta5, Ti40-Ta5, and Ti80-Ta5.

### 2.2. Sample Characterization

A scanning electron microscope (SEM) (Hitachi SU8010, Hitachi High-Technologies Co., Tokyo, Japan) and atomic force microscope (AFM) (Park NX10, Park Systems Corp., Suwon, Republic of Korea) were used to examine the surface morphology of the samples. The quantitative analysis of AFM images, including surface roughness parameters, such as the average surface roughness (Sa) and the root mean square roughness (Sq), was performed. 

Contact angle measurements on Ti discs were performed thrice in triplicate using the DSA 100 Goniometer (A.KRÜSS Optronic GmbH, Hamburg, Germany) equipped with an automatic liquid drop dosing system. A 4 μL droplet of distilled water was dropped over titanium discs. The contact angle was measured from the right and left sides of the droplet on the titanium disc surface. In total, 3 measurements were obtained for each sample and the contact angle was calculated as the mean of all measurements. The contact angle measurements were taken directly after the sample LTP treatment of the Ti discs was completed.

In order to analyze the chemical composition of the surface of the samples, X-ray photoelectron spectroscopy (XPS) was performed. Measurements were carried out in ultra-high-vacuum conditions (base pressure in the analysis chamber was ≤2 × 10^−10^ mbar. The source of excitation radiation was a standard AlKα lamp (hν = 1486.6 eV). The angle between the excitation beam and the surface normal of the samples was 55 degrees. The energy of the photoelectron spectra was recorded using a hemispherical VG-Scienta R3000 analyzer (Scienta Omicron, Uppsala, Sweden). The energy step was set to ΔE = 0.1 eV. The experimental data were fitted to Gauss–Lorentz shapes using CasaXPS software (Version 2.3.16) (Casa Software Ltd., Teignmouth, UK).

Ellipsometric azimuths Ψ and Δ for the Ti-6Al-4V substrate were measured in the spectral range from 0.6 eV (2066 nm) to 5.0 eV (248 nm). Measurements for tannic acid-modified samples were made in the 0.6–3.0 eV (2066–413 nm) spectral range. All of the ellipsometric measurements were made for three angles of incidence (65°, 70°, and 75°) using the V-VASE instrument (J.A. Woollam Co., Inc., Lincoln, NE, USA). Apart from Ψ and Δ azimuths, the depolarization factor (%Depol) was registered. The WASE32 software (version 3.774) (J.A. Woollam Co., Inc., Lincoln, NE, USA) was used to analyze the ellipsometric data.

To determine the optical constants of the bare Ti-6Al-4V substrate, the substrate\ambient optical model was used. The effective complex refractive index ($\tilde{n}$) of the substrate was described using the following formula including a Drude term and the sum of Lorentzian oscillators [40,41]:

(1) $${\tilde{n}}^{2}={\left(n+ik\right)}^{2}=\varepsilon_{\infty }-\frac{{\left(\hbar \omega_{p}^{\;}\right)}^{2}}{E^{2}-E\hbar \Gamma }+\sum_{j=1}^{m}\frac{A_{j}E_{j}^{2}}{E_{j}^{2}-E_{\;}^{2}+i{Br}_{j}E},$$

where *n* and *k* are the real part of $\tilde{n}$ and the extinction coefficient, respectively. In Equation (1), $\varepsilon_{\infty }$ (set to 1) is the high-frequency dielectric constant, $\hbar \omega_{p}$ is the plasma energy, ℏΓ is the Drude broadening related to the relaxation of free carriers, while $A_{j}$, $E_{j}^{\;}$ and ${Br}_{j}$ are the amplitude, energy, and broadening of the *j*-th oscillator.

The thickness of tannic acid films was determined using the four-medium optical model of a sample (the Ti-6Al-4V substrate\graded intermix\tannic acid\ambient). The optical constants of the Ti-6Al-4V substrate were established in a separate experiment. The tannic acid is transparent in the NIR-vis spectral range. Therefore, the refractive index was parameterized using a pole dispersion relation, which is an equivalent of the Sellmeier dispersion relation [40,41]:

(2) $$n^{2}=\frac{A_{0}}{E_{0}^{2}-E^{2}},$$

where $E_{0}^{\;}$ is the pole oscillator position and $A_{0}$ is its magnitude. The graded intermix layer was modeled as a set of ten Bruggeman Effective Medium Approximation (BEMA) layers, where the fraction percentage of the Ti-6Al-4V substrate changes from 100% to 0% (and appropriately, the fraction percentage of the tannic acid increases from 0% to 100%). The shape of the depth profile was chosen as exponential (symmetric). The mathematical formulas were implemented in the WASE32 software (version 3.774) [40]. The optical properties of all tannic acid films are the same; therefore, to establish the dispersion parameters of Equation (2) (and thus the dispersion relation of *n* for the acid) and thicknesses of the intermix (*d_i_*) and acid layers (*d*), the multiple-sample analysis was applied [40,41,42,43,44,45]. In this approach, the refractive index of the tannic acid was assumed to be the same for all the layers produced, while thicknesses were determined separately for all of the samples. It should be noted that the depolarization of the reflected light was observed during the ellipsometric measurements; therefore, the percentage of depolarized light (*%Depol*) was determined using the following equation [40,41]:

(3) $$\%Depol=100\%\left(1-\alpha^{2}-\beta^{2}-\gamma^{2}\right),$$

where $\alpha =\cos\left(2\Psi \right)$, $\beta =\sin\left(2\Psi \right)\cos\left(\Delta \right)$, and $\gamma =\sin\left(2\Psi \right)\sin\left(\Delta \right)$. This phenomenon originates from the layer thickness non-uniformity or can be caused by patterned substrates. Based on the depolarization factor (*%Depol*), we estimated the thickness non-uniformity (*N_d_*) of the tannic acid coatings.

## 3. Results and Discussion

**Figure 1:**
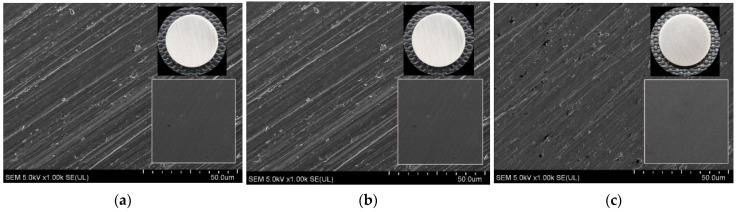
SEM images of the surface of titanium alloy discs (left to right): Ti0 (**a**), Ti40 (**b**), Ti80 (**c**); insets: macroscopic photo images of the investigated discs (upper right), SEM image of the surface of titanium alloy discs at 50× magnification (lower right); acceleration voltage: 1.0 kV; width: 1.0 mm.

**Figure 2:**
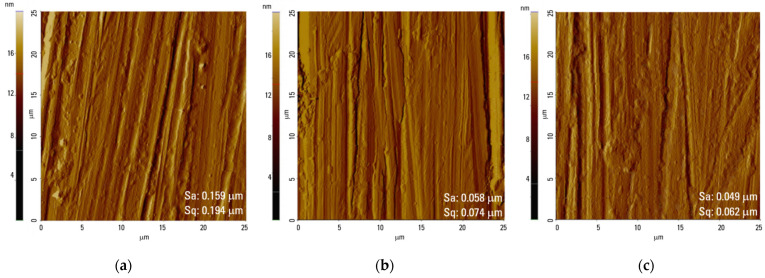
AFM height images of the surface of titanium alloy discs (left to right): Ti0 (**a**), Ti40 (**b**), Ti80 (**c**); values of average surface roughness (Sa) and root mean square (Sq) roughness.

**Figure 3:**
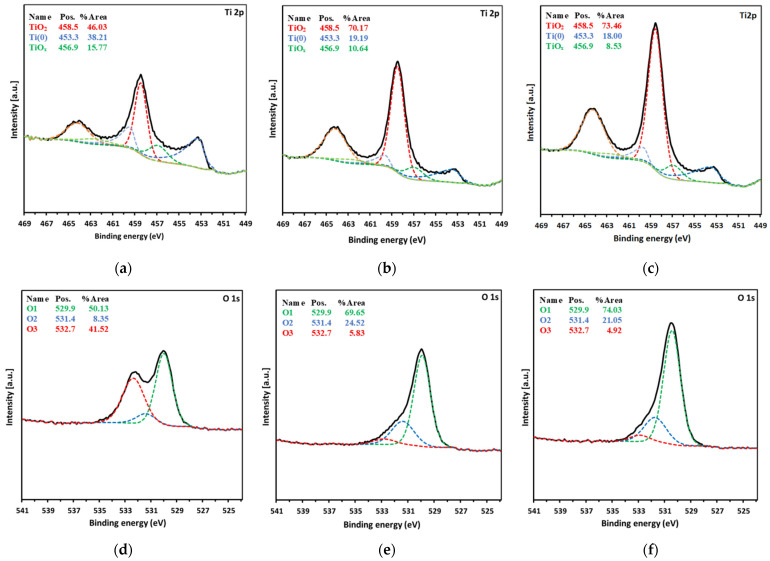

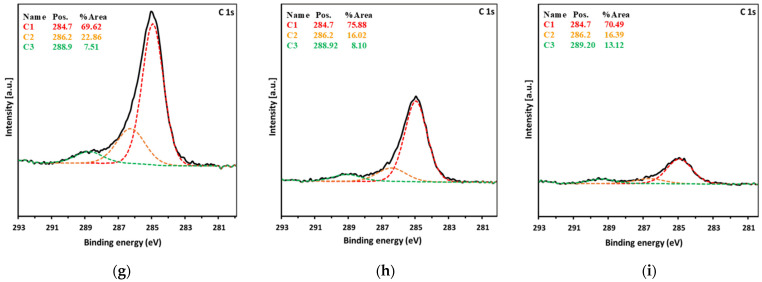
XPS spectra of Ti2p level (**a**–**c**), O1s level (**d**–**f**), and C1s level (**g**–**i**) presented in columns (left to right) for Ti0 discs, Ti40 discs, and Ti80 discs, respectively.

The surface morphology of the Ti0, Ti40, and Ti80 discs is displayed in Figure 1 (SEM images) and Figure 2 (AFM images). The chemical compositions of the surfaces of the Ti0, Ti40, and Ti80 discs, gained from the XPS measurements, are listed in Table 1, whereas Figure 3 depicts the deconvoluted XPS spectra of the Ti2p level (Figure 3a–c), O1s level (Figure 3d–f), and C1s level (Figure 3g–i), respectively.

From the SEM observations, it can be seen that all the samples exhibit very similar topography characterized by parallel grooves and ridges originating from the preceding polishing with abrasive SiC paper. There are numerous pits detected on the untreated surface (Figure 1a and Figure 2a), the number of which is reduced on the plasma-treated samples (Figure 1b,c and Figure 2b,c). From the SEM and AFM images, it can be noted that grooves seen on the LTP-treated surfaces are more shallow, whereas the ridges are smoother compared to the reference surface. The values of surface roughness (Sa) and the root mean square (Sq) roughness are slightly lower for Ti40 and Ti80 compared to the reference Ti0 discs. No significant effect on the morphology changes was found resulting from the applied power of LTP modification.

The analysis of the registered spectra of the Ti2p level for the Ti0 discs (Figure 3a) shows that about 46% of the spectrum area of this level is responsible for photoelectrons from titanium atoms forming TiO_2_—the peak is at the energy of 458.5 eV. The characteristic asymmetric peak coming from metallic titanium was recorded at an energy of 453.3 eV [46]. Its intensity compared to the others is about 38%. The remaining 16% of the surface area of the Ti2p level consists of the peak observed at 456.9 eV originating from an oxidation state of Ti_2_O_3_. The LTP modification changes these proportions—the content of metallic titanium drops to 19% and 18% for the Ti40 discs and Ti80 discs, respectively, while the TiO_2_ concentration increases to 70% and 73% for the Ti40 discs and Ti80 discs, respectively (Figure 3b and Figure 3c, respectively).

The atomic surface concentration of titanium was approximately 10% on the Ti0 discs and increased to approximately 17% and 24% after oxygen plasma surface modification in the Ti40 discs and Ti80 discs, respectively. When comparing the LTP-treated discs to the untreated discs, the surface atomic concentration of titanium atoms attributed to TiO_2_ (circa 5%) increases as a result of the applied modification and increases with the applied power of the modification (circa 12% for Ti40 discs and circa 18% for Ti80 discs). The atomic concentration of titanium atoms attributed to metallic titanium and Ti_2_O_3_ (circa 3–4% and less than 2%, respectively) remains at a similar level, which implies that the applied LTP modification induces the formation of TiO_2_.

The O1s level of oxygen shown for Ti0 discs in Figure 3d contains three components. The main component, marked as O1, is the peak with an energy of 529.9 eV that corresponds to O_2_, which originates from double-bonded oxygen pertaining to the titanium oxides, Ti_2_O_3_ and TiO_2_. The peak at an energy of 531.4 eV marked as O_2_ belongs to oxygen atoms bound to hydroxyl groups, whereas the peak with an energy of 532.7 eV marked as O3 depicts oxygen atoms bound to carboxyl groups [47,48]. The Ti0 discs are clearly distinguished by the high intensity of the latter peak (approx. 42%) in comparison with the spectra of LTP-modified samples, where the content of this peak in the O1s level drops to the level of 6% and 5% for Ti40 discs and Ti80 discs, respectively. Due to the applied plasma treatment, the intensity of the peaks attributed to titanium oxides and hydroxyl groups increases from circa 50% in Ti0 discs to circa 70% in Ti40 discs and 75% in Ti80 discs, and from circa 3% in Ti0 discs to circa 9% in both Ti40 discs and Ti80 discs. 

The atomic surface concentration of oxygen is about 32% in the Ti0 discs and increases to about 40% and 54% after the oxygen plasma surface modification in the Ti40 discs and Ti80 discs, respectively. When comparing LTP-treated discs to untreated discs, the surface atomic concentration of oxygen atoms pertaining to TiO_2_ (16%) increases as a result of the applied modification and increases with the applied power of the modification (circa 28% for Ti40 discs and 40% for Ti80 discs). Furthermore, the concentration of oxygen atoms bound to hydroxyl groups increases with LTP treatment and is reflected at a level of approximately 10% and approximately 11% for Ti40 discs and Ti80 discs, respectively, referred to as approximately 3% in the case of Ti0 discs. On the other hand, the oxygen assigned to carboxyl groups decreased from about 13% in the Ti0 discs to about 2% in both discs that underwent the LTP treatment.

The deconvolution of C1s spectra reveals three components. The main component is the peak with an energy of 284.7 eV, which corresponds to C–C and/or C–H bonds. The peak with an energy of 286.5 eV belongs to a C–O bond, whereas the peak with an energy of 288.9 eV can be assigned to an O–C=O bond [49,50]. It is likely that due to partial oxidation, carbon contamination has been removed as CO or CO_2_. The distribution of individual factions of C undergoes slight changes, and no significant or consistent trends are observed.

The surface of Ti0 discs shows a higher carbon concentration (circa 48%) than Ti40 and Ti80 discs and a significant difference depending on the LTP exposure power is visible in the surface atomic concentration of carbon. It drops to about 38% in the Ti40 discs and to about 15% in the Ti80 discs. The higher participation of carbon in the whole surface composition of the Ti0 samples can be attributed to the adventitious carbon usually found on the surface of most air-exposed samples. The observed relation coincides with the thicker layer of surface oxides generated due to the oxidative treatment of the disc.

On the surface of the investigated discs, Al, Si, and N were also identified. Their presence was not subjected to in-depth analyses. The origin of Al is Ti-6Al-4V. The presence of N (approx. 2.7% for the Ti0 discs and approximately 1.5–2% for the discs subjected to LTP) is explained as possibly being introduced from the air after the modification process. Si was identified as the remaining SiC abrasive material used in the preparations of the discs and was detected only in the untreated Ti0 discs, which means that the LTP process cleans the surface of the Si residues found.

**Figure 4:**
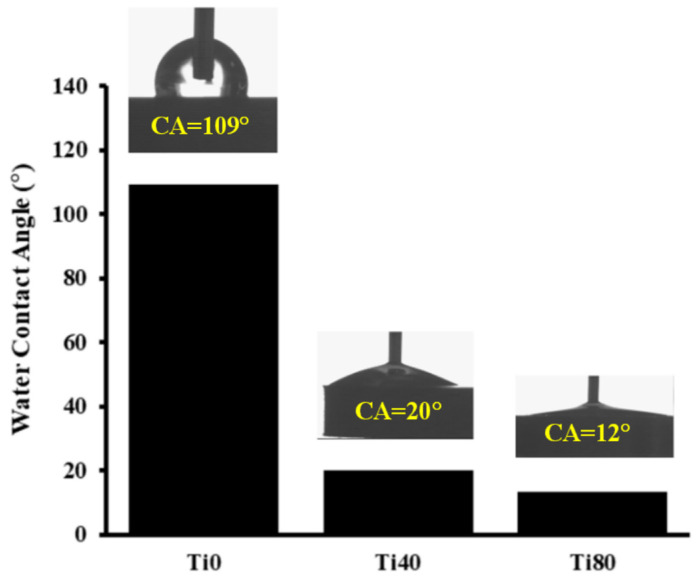
Contact angle of Ti0 vs. Ti40 and Ti80 discs.

Significant differences were found in the contact angles between the untreated Ti substrate (Ti0) and the oxygen plasma-modified discs (Ti40 and Ti80) (Figure 4). The water contact angle of the Ti0 discs was 109°, while after plasma treatment, it dropped to 20° and 12° for the Ti40 discs and the Ti80 discs, respectively.

**Figure 5:**
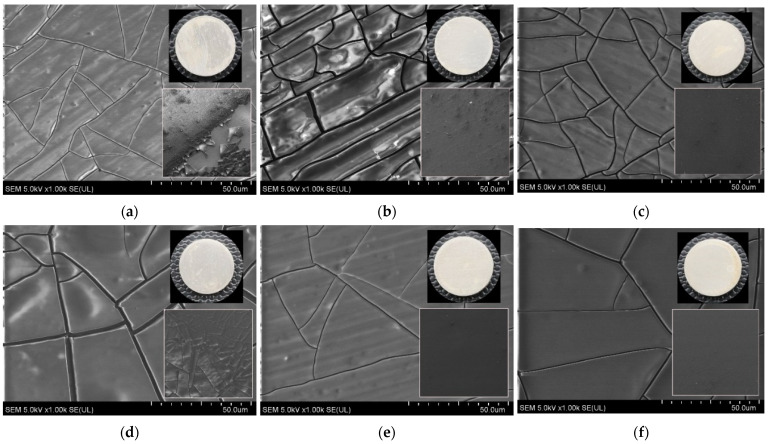
SEM images of the surface of the tannic acid coating deposited on titanium alloy discs using tannic acid with a concentration of 2% (left to right): Ti0-Ta2 (**a**), Ti40-Ta2 (**b**), and Ti80-Ta2 (**c**); using tannic acid with a concentration of 5% (left to right): Ti0-Ta5 (**d**), Ti40-Ta5 (**e**), and Ti80-Ta5 (**f**); insets: macroscopic photo images of the investigated discs (upper right), SEM images of the surface of tannic acid coatings deposited on titanium alloy discs at 50× magnification (lower right); acceleration voltage: 1.0 kV; width: 1.0 mm.

**Figure 6:**
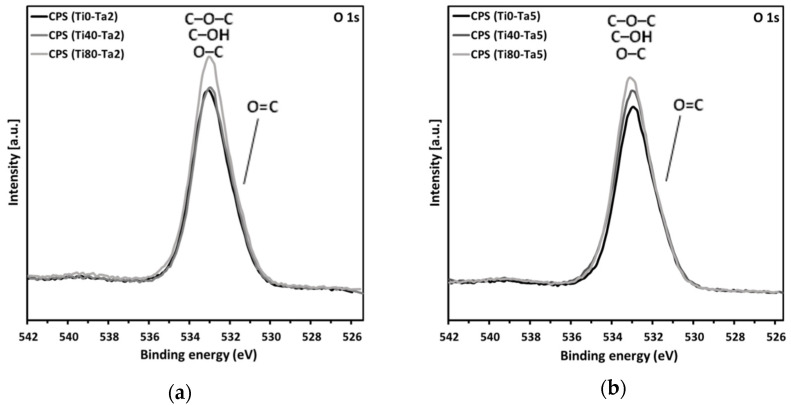

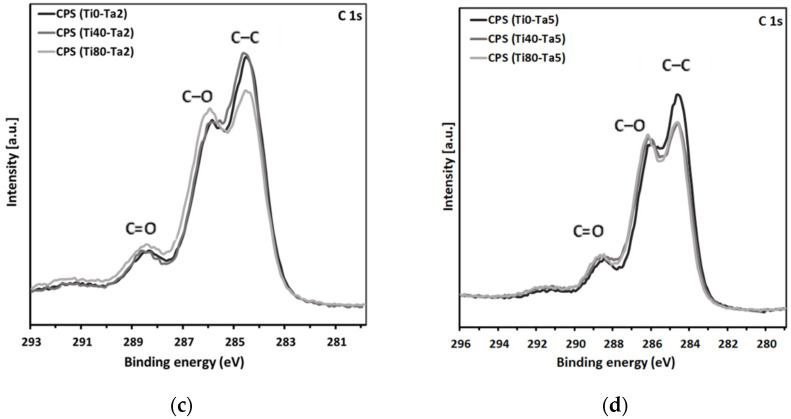
XPS spectra of the O1s level and the C1s level recorded for the Ti0-Ta2, Ti40-Ta2, and Ti80-Ta2 discs (**a**,**b**) and Ti0-Ta5, Ti40-Ta5, and Ti80-Ta5 discs (**c**,**d**).

Figure 5 shows SEM images of the surface microstructure of the tannic acid coatings deposited on the titanium alloy discs. The chemical composition of the surface of the Ti0-Ta2, Ti40-Ta2, and Ti80-Ta2 discs and the Ti0-Ta5, Ti40-Ta5, and Ti80-Ta5 discs, gained from the XPS measurements, are listed in Table 2 and Table 3, respectively, whereas Figure 6 depicts the XPS spectra of the O1s level (Figure 6a,b) and the C1s level (Figure 6c,d).

From the SEM images, it can be seen that all of the discs are covered with an evenly distributed tannic acid layer. The visible cracks on the surface are the result of drying shrinkage. The tannic acid coatings on the surface of untreated Ti are delaminating, which is manifested by numerous protrusions and bulges along the cracks in the case of Ti0-Ta2 (Figure 5a), while the surface of Ti0-Ta5 additionally has thicker cracks (Figure 5a). For both discs Ti0-Ta2 and Ti0-Ta5, the lighter areas of the coatings are interpreted as areas that are loose or not fully bonded to the substrate. The delamination is evidently seen at the inset showing the surface of Ti0-Ta2 (Figure 5a). A similar effect of partial delamination of the coating from the Ti substrate occurs on the Ti40-Ta2 disc. The SEM image of these Ti40-Ta2 discs (Figure 5b) shows numerous protrusions and bulges along the cracks. Furthermore, for Ti40-Ta2 discs (Figure 5b), a higher number of cracks occurs within the coating compared to other discs, and these cracks are parallel with grooves remaining during the preceding polishing of the titanium alloy surface. The observation of the SEM images also allows us to assess that in discs Ti80-Ta2, Ti40-Ta5, and Ti80-Ta5, the TA coating is well bonded with the substrate, as the SEM images displaying the surface of the particular coatings (Figure 5c, Figure 5e and Figure 5f, respectively) are homogenous without defects, indicating delamination or incomplete bonding to the substrate, and have thinner cracks. The SEM images of Ti40-Ta5 (Figure 5c) and Ti80-Ta2 (Figure 5e) reveal parallel grooves and ridges originating from the preceding polishing visible through the coating, while with the SEM image of the Ti80-Ta5 disc, such a substrate morphology is not revealed, which can be interpreted as the coating on the Ti80-Ta5 disc being thicker compared to Ti40-Ta5 and Ti80-Ta2.

In the case of all samples, the survey spectra did not show the presence of elements other than tannic acid components—i.e., carbon and oxygen—on the surface. At the C1s level, four characteristic components of tannic acid are visible. The peak at about 284.7 eV, interpreted as coming from C–C and C–H bonds, had the highest intensity (about 50% of the area of the C1s level) [50]. At about 286.3 eV, there is a clear peak (ca. 40%) associated with the C–O group. At 288.7 eV, there is a C=O bond peak, and at 291.5 eV, there is a weak π-π* shake-up satellite peak characteristic of aromatic rings. The proportion of C–O slightly increases in the case of samples covered with 5% TA as compared with 2% TA. 

The oxygen O1s level can be adjusted with two components. C–O–C, C–OH, and O–C bonds account for the peak at 533.3 eV. The peak at 531.8 eV is interpreted to be coming from O=C bonds.

**Figure 7:**
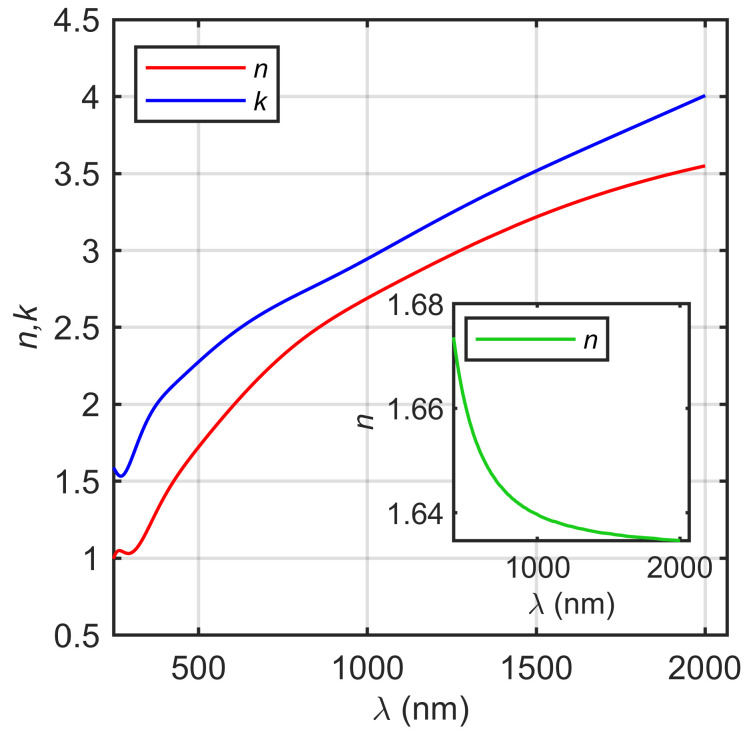
The effective refractive index and the extinction coefficient of the Ti-6Al-4V substrate. Inset: the refractive index of the tannic acid layer.

In Figure 7, the determined *n* and *k* effective spectra are presented. The shape of the spectra is typical for conducting materials, i.e., the strong absorption in the near-infrared (NIR) (the Drude term) and interband transitions in the ultraviolet (UV) and visible (vis) spectral ranges [42,43,51,52]. It should be noted that the optical data are effective quantities, including all of the nonidealities of the substrate (native oxides and roughness).

The determined thickness of the intermix (*d_i_*) and tannic acid (*d*) layers and the thickness non-uniformity of the tannic acid coating (*N_d_*) are summarized in Table 4. During the analysis of ellipsometric data, we assumed that the optical constants of the intermix layer are gradually changing from the optical constants established for the substrate (see Figure 7) to the optical constants of the tannic acid. The reason for this approach was caused by the state of the Ti-6Al-4V substrate both before and after plasma modification. The determined thickness of the intermix layer is in the range from about 550 to about 840 nm. The thickness of tannic acid increases with the plasma power from 526 ± 4 nm (Ti0-Ta2) to 894 ± 7 nm (Ti40-Ta2) to 2189 ± 20 nm (Ti80-Ti2) and from 686 ± 6 nm (Ti0-Ta5) to 1773 ± 20 nm (Ti40-Ta5) to 2708 ± 24 nm (Ti80-Ta5). The SEM images of the produced samples (see Figure 5) show their non-uniform covering and cracks on their surfaces. Therefore, apart from the Ψ and Δ azimuths, during the ellipsometric measurement, the depolarization factor was registered [40,41]. Based on these results, the thickness non-uniformity (*N_d_*) parameter was determined. The estimated values of *N_d_* for all tannic acid layers are in the range of 27% to 57% (see Table 4), which confirms the SEM observations. The refractive index of the tannic acid exhibits a normal dispersion and decreases from 1.67@413 nm to 1.63@2066 nm (see the inset in Figure 7).

## 4. Conclusions

This study aimed to investigate the effect of the modification of a titanium alloy surface by low-temperature oxygen plasma on the deposition of a tannic acid coating. The structural and morphological properties of the Ti-6Al-4V substrate without and with the LTP treatment applied were comprehensively examined, and the impact of the LTP exposure power increase on these properties was studied. Following this, the tannic acid coatings were deposited on the titanium alloy surface and the structural and morphological properties of the deposited tannic acid coating were examined.

Based on the results of the XPS analysis of the titanium alloy substrate, it was observed that LTP treatment leads to the formation of titanium dioxides containing –OH groups on the surface, which is justified by an increase in the amount of titanium in the oxidation state Ti^4+^ and an increase in the amount of oxygen double bonded with Ti and hydroxyl groups. These are accompanied by a reduction in carbon, which indicates a pollutant on the substrate. The contact angle of the titanium substrate was significantly reduced due to LTP treatment, and it increased with the applied plasma power. The superior hydrophilicity and significantly high surface wettability of the LTP-treated Ti alloy substrate are explained by increased hydrophilic –OH groups and decreased hydrophobic hydrocarbons on the LTP-modified surface.

The observed improvement in the properties of the low-temperature plasma-modified titanium alloy substrate is attributed to the deposited tannic acid coating. For the LTP-treated titanium substrate, there was a perceived better quality of the coatings, which stood out due to their higher uniformity, lower number of defects indicating delamination or incomplete bonding, lower number of cracks, thinner cracks, and higher thickness of the coatings.

The results show that applying titanium alloy surface oxidation through oxygen plasma treatment enhances conditions for tannic acid deposition, and the applied plasma power affects the coating quality.

## Figures and Tables

**Table 1:** XPS results: surface composition of the Ti0 discs, Ti40 discs, and Ti80 discs.

Spectrum	eV	Assignment of Peak	Ti0 at. %	Total Content	Ti40 at. %	Total Content	Ti80 at. %	Total Content
Ti2p	453.3	Ti(0)	1.61	10.23	1.78	16.69	2.07	24.22
	456.9	Ti_2_O_3_	3.91		3.20		4.36	
	458.5	TiO_2_	4.71		11.71		17.79	
O1s	529.9	O1	15.99	31.89	28.17	40.45	40.00	54.04
	531.4	O2	2.66		9.92		11.38	
	532.7	O3	13.24		2.36		2.66	
C1s	284.7	C–C, C–H	33.69	48.42	29.01	38.24	10.63	15.08
	286.2	C–O	11.07		6.13		2.47	
	288.9	O–C=O	3.64		3.10		1.98	
N				2.67		1.53		2.06
Al				1.58		3.09		4.60
Si				5.23		0.00		0.00
Total				100.00		100.00		100.00

**Table 2:** XPS results: surface composition of the Ti0-Ta2 discs, Ti40-Ta2 discs, and Ti80-Ta2 discs.

Spectrum	eV	Assignment of Peak	Ti0-Ta2 at. %	Total Content	Ti40-Ta2 at. %	Total Content	Ti80-Ta2 at. %	Total Content
C1s	284.7	C–C	38.66	71.47	36.84	71.09	39.52	71.60
	286.3	C–O	25.49		26.98		25.50	
	288.7	C=O	7.32		7.27		6.58	
O1s	531.8	O=C	5.02	28.53	4.95	28.91	5.52	28.40
	533.3	C–O–C, C–OH, O–C	23.51		23.96		22.88	
Total				100.00		100.00		100.00

**Table 3:** XPS results: surface composition of the Ti0-Ta5 discs, Ti40-Ta5 discs, and Ti80-Ta5 discs.

Spectrum	eV	Assignment of Peak	Ti0-Ta5 at. %	Total Content	Ti40-Ta5 at. %	Total Content	Ti80-Ta5 at. %	Total Content
C1s	284.7	C–C	30.96	68.19	31.17	67.99	30.83	67.06
	286.3	C–O	29.24		29.65		28.80	
	288.7	C=O	7.99		7.17		7.43	
O1s	531.8	O=C	3.58	31.81	3.30	32.01	4.10	32.94
	533.3	C–O–C, C–OH, O–C	28.23		28.71		28.84	
Total				100.00		100.00		100.00

**Table 4:** Thickness of the intermix (*d_i_*) and tannic acid (*d*) layers and thickness non-uniformity of the tannic acid film (*N_d_*).

Sample	*d_i_* (nm)	*d* (nm)	*Nd* (%)
Ti0-Ta2	837 ± 23	526 ± 4	29 ± 2
Ti40-Ta2	573 ± 134	894 ± 7	44 ± 2
Ti80-Ta2	546 ± 54	2189 ± 20	57 ± 2
Ti0-Ta5	560 ± 116	686 ± 6	40 ± 2
Ti40-Ta5	672 ± 278	1773 ± 20	34 ± 2
Ti80-Ta5	614 ± 35	2708 ± 24	27 ± 1

## Data Availability

The data presented in this study are available on request from the corresponding author.
